# Modulation of executive attention by threat stimulus in test-anxious students

**DOI:** 10.3389/fpsyg.2015.01486

**Published:** 2015-10-01

**Authors:** Huan Zhang, Renlai Zhou, Jilin Zou

**Affiliations:** ^1^Key Laboratory of Child Development and Learning Science of Ministry of Education, Research Center for Learning Science, Southeast University, NanjingChina; ^2^School of Public Administration, Wuhu Institute of Technology, WuhuChina; ^3^Department of Psychology, School of Social and Behavioral Sciences, Nanjing University, NanjingChina; ^4^State Key Laboratory of Cognitive Neuroscience and Learning, BeijingChina; ^5^Beijing Key Laboratory of Applied Experimental Psychology, School of Psychology, Beijing Normal University, BeijingChina

**Keywords:** test anxiety, emotional distraction, attention network test, executive attention, modulation

## Abstract

The study examined whether test anxiety (TA) is related to impaired attentional networks under emotional distraction. High and low test-anxious students completed a modified version of the attention network test (ANT) in which emotional distracters, specifically threat-related or neutral words, were embedded in centrally presented hollow arrows in Experiment 1. Results showed a significant reduction in efficiency of the executive attention in test-anxious students compared to controls when the fillers were threat/test-related words. To evaluate the effect of the test adaptation, the original ANT, which utilized no emotional distracter, was employed as a control task in Experiment 2. We then consolidated the data on efficiency of attentional networks, which were derived from both tasks. Contrasting the two tasks showed that TA reduced executive attention in the revised task only, suggesting an enhanced sensitivity provided by the adaptation from the original task. Taken together, these findings indicate that the attentional deficit in test-anxious individuals represents a situation-related defect of a single component of attention rather than an underlying structural and universal attentional deficit. The results support the hypothesis of attentional control theory and contribute to the understanding of attentional mechanisms in individuals with TA.

## Introduction

Test anxiety (TA) has been described as a set of phenomenological, physiological, and behavioral responses that accompany concerns about possible negative consequences or failure in an exam or similar evaluative situations (Zeidner, 1998). It has been found that TA was related to susceptibility or attentional bias to threat distraction (Mathews, 1993; Keogh and French, 2001; Putwain et al., 2011). Hence, TA was deemed to induce a type of information processing (Zeidner, 1998), concretely, attentional deficit (Calvo et al., 1994). Considering that attention has been researched as a unitary idea in the past (Raz and Buhle, 2006), it is not entirely clear whether susceptibility or bias to threat-related distracters is due to underlying structural and universal deficits of attention or due to situational defect of a single component of attention.

It is important to note that attention characteristics in test-anxious individuals have been examined in a variety of studies using different paradigms, such as the dot probe task (Putwain et al., 2011), cue-target task (Keogh and French, 2001; Liu et al., 2015), central cue task (Chen et al., 2011), negative priming task (Shi et al., 2014), Stroop task (Hopko et al., 2002; Kofman et al., 2006; Lawson, 2006; Bradley et al., 2010; Geen and Kaiser, unpublished manuscript), and switching task (Kofman et al., 2006). The results from these tests are hard to compare directly due to different aspects of attention that were tested by the different tasks (Miyake et al., 2000; Joormann, 2004; Derrfuss et al., 2005; Mottaghy et al., 2006; Mogg et al., 2008; Finucane et al., 2010). Therefore, it is critical to employ a comprehensive and systematic measurement to inspect potential attentional deficits in test-anxious people thoroughly.

Among the various instruments to assess attention characteristics, the attention network test (ANT) is a popular tool that was designed as a quick and simple computerized task based on a neural network model of attention (Posner and Petersen, 1990; Fernandez-Duque and Posner, 2001; Posner et al., 2007; Petersen and Posner, 2012). According to this model, the attention system of the human brain can be divided into three functionally and anatomically independent networks, each with corresponding functions. For example, the alerting network allows producing and maintaining optimal vigilance; the orienting network is focused on the ability to prioritize sensory input by selecting a modality or location; and the executive control network allows for the monitoring and resolution of conflict between responses. These networks used to be analyzed separately until a single task (ANT) was created through the combination of a flanker paradigm and cueing task with fully randomized conditions within blocks (Dennis et al., 2008; Macleod et al., 2010). Participants were instructed to press either the left or right key of the keyboard (or mouse) depending on whether the target, a central arrow presented above or below the fixation point, pointed to the left or the right, respectively. Through the integration of separate chronometric analyses for each attention network, the ANT can tap into early and late stages (components) of information processing (Raz and Buhle, 2006; Galvao-Carmona et al., 2014). It has been widely accepted as a useful, accurate, and reliable measure of function of the three attentional subsystems in light of the evidence from various behavioral and brain imaging studies (Petersen and Posner, 2012). The ANT was also applied to explore individual differences in Caucasians (Matthews and Zeidner, 2012), and Chinese (Du et al., 2006).

Recently, four versions of the emotional ANT have been developed to explore the effects of emotional distracters on attentional functions. In earlier versions, each trial was preceded by an emotional picture presented for 50 ms (Dennis and Chen, 2007), or every sixth trial was preceded by an emotional picture presented for 6 s (Finucane et al., 2010). In more recent versions, the cues (asterisks) were replaced by emotional pictures (Cohen et al., 2011) or words (Gómez-Íñiguez et al., 2014). All of these tasks are useful, as they provide insight into the effect of emotion on attentional processing. However, we are skeptical of their sensitivity to differentiate between high and low test-anxious people under emotional distraction. The main reason for this insensitivity may lie in the time lag between emotional and target processing, which can help test-anxious people to summon extra resources and thus compensate for potential performance impairments caused by the resource preemption of emotional processing (Eysenck et al., 2007). More concretely, the extra resources may compensate for depleted self-control resources which help test-anxious people regulate the disruptive effects of emotional stimuli on subsequent cognitive processes (Cohen et al., 2011; Bertrams et al., 2013).

**FIGURE 1:**
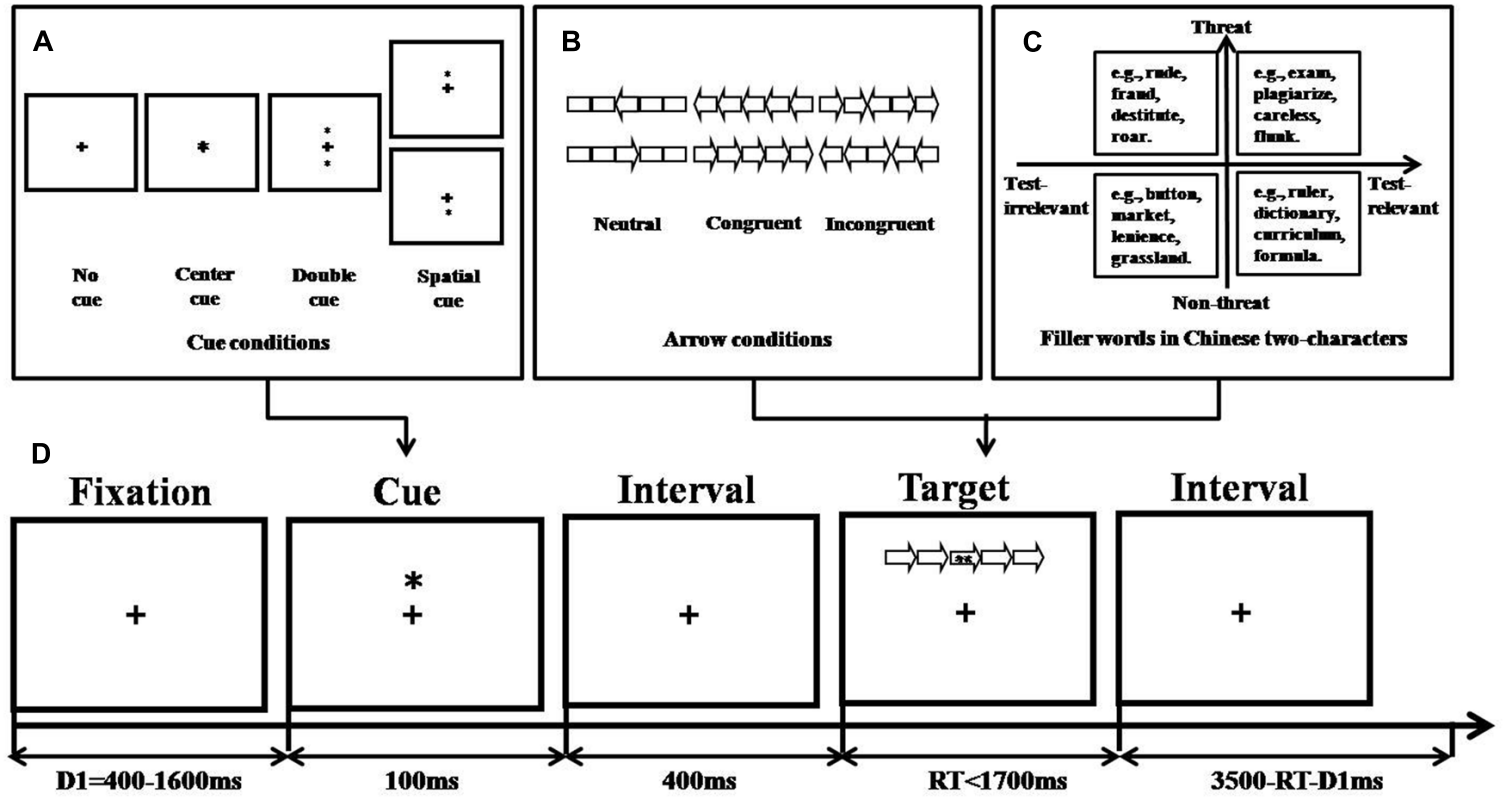
**Experimental procedure of revised ANT (based on Fan et al., 2002). (A)** Cue conditions, **(B)** Arrow conditions, **(C)** Filler word conditions, and **(D)** An example of the procedure.

In the present study, we developed a novel version of the ANT by embedding a task-irrelevant distracter (Chinese two-character word differing in valence and relevance to the examinations) into the target (central hollow arrow). Due to the new, yet the same, perceptual object which was formed by the distracter and target (Berti and Schröger, 2001), participants were forced to attend to the distracter which was presented in the whole object (Leiva et al., 2015); consequently, visual distraction effects were obtained (Berti and Schröger, 2004). The adaptation allows us to induce simultaneous competition for attention resources between the target and emotional distracter and serves as a possible source of emotional interference (illustrated in **Figure 1**). More importantly, TA individuals had no supplemental resources in our modified ANT compared to the previous revised ANTs, due to the simultaneous presentation of distracters and targets, to compensate for their potential performance impairments caused by the emotional distraction. Hence, our modified ANT might tap into the effect of TA on attentional functions.

The aim of this study was twofold. First, we examined whether TA is related to impaired attentional networks under emotional distraction. In Experiment 1, we employed the revised ANT to explore the effects of emotional distraction on attentional functions. Second, we assessed the validity of this revised task to measure attentional networks in high versus low test-anxious students. For this purpose we proceeded in two steps: (i) replicating the original ANT in Experiment 2, and (ii) comparing the data on efficiency of attentional networks, which were recorded in the two different situations, that is, derived from the original (conditions free from emotional distracters) and revised tasks (conditions under emotional distracters).

Hypothesis 1: Threat/test-related distracters embedded in targets are expected to induce stronger attention interference effects (i.e., lower efficiency of alerting, orienting, and executive attention) in contrast to neutral distracters in high versus low test-anxious students.

## Experiment 1

### Methods

#### Participants

Forty (19 males) college students (see **Table 1**) were selected from a total of 422 (197 males), aged 18–26 years (*M* = 20.38, *SD* = 1.12), according to their scores of the Chinese version of the Test Anxiety Inventory (TAI; Spielberger, 1980; Wang, 2003). Those falling into the highest and lowest quarters of the score distributions were selected as the 40 candidates (high-anxious students and controls, respectively). All participants (native Chinese speakers) reported normal or corrected-to-normal vision and right-handedness. None of them suffered from psychosis, neuropathy or had experienced drug abuse. They took no medications or alcohol in the 3 days before the experiment. All participants signed the written informed consent, and they were paid for their participation. The study was approved by the local ethics committee.

**Table 1:** Demographic characteristic of subjects in the two experiments.

Task	Group	Gender (N)		Age *M* (*SD*)	TA *M* (*SD*)	Trait anxiety *M* (*SD*)	State anxiety *M* (*SD*)	Depression *M* (*SD*)
		Male	Female					
**(A)** Revised ANT	Control	10	10	20.45 (0.95)	25.10 (2.51)	38.70 (8.02)	35.50 (5.31)	3.50 (3.36)
	Experiment	9	11	20.20 (1.06)	47.75 (5.88)	49.60 (6.92)	44.85 (7.31)	9.60 (4.94)
**(B)** Original ANT	Control	9	10	19.89 (0.88)	25.39 (2.64)	38.37 (7.93)	33.63 (7.31)	2.79 (2.02)
	Experiment	10	11	19.90 (0.94)	49.65 (8.25)	50.71 (7.71)	42.33 (7.37)	10.67 (5.09)

#### Measures

##### Test anxiety

The Chinese version of the 20-items TAI (Wang, 2003), including the two concepts worry and emotionality, was used to measure TA. On a four-point scale, participants rated the frequency with which they experienced specific symptoms of anxiety before, during, and after exams. Cronbach’s alpha of the Chinese Version of the TAI (total) in this study (0.90) was comparable with that (0.92) reported in a previous study (Spielberger, 1980); the corresponding values for the worry and emotionality subscale were 0.80 and 0.84, respectively (Wang, 2003).

##### State anxiety and trait anxiety

The State-Trait Anxiety Inventory (STAI; Spielberger, 1983), including the A-Trait and A-State subscale, was used to measure trait and state anxiety separately. On a four-point scale, participants rated the frequency of specific symptoms they experienced before in the A-Trait subscale or rated the correspondence between the descriptions and their current feelings in the A-State subscale. A score of 20 indicates the absence of anxiety and a score of 80 indicates high anxiety in each subscale. The Chinese version of the STAI (Tsoi et al., 1986), showing high internal consistency, was used in the present study. Shek (1988) reported that the Cronbach’s alpha was 0.90 and 0.81 for A-State and A-Trait subscale, respectively.

##### Depression

The short form of the Beck Depression Inventory (BDI; Beck et al., 1974, 1988) was used to measure the respondent’s current depressive state on a four-point scale. The inventory taps symptoms and attitudes frequently displayed by depressed psychiatric patients and relatively infrequently by non-depressed psychiatric patients (Beck et al., 1961). The Chinese version of the BDI (Chan and Tsoi, 1984) was used in the present study. The overall Cronbach’s alpha was 0.86 (Shek, 1990), indicating good reliability.

##### Vocabulary assessment

One thousand and forty-six two-character Chinese words (including 476 nouns, 359 verbs, and 211 adjectives) with frequency counts below 1000 were chosen from a frequency list of a 20-million word corpus of the Chinese Linguistics Data [DB/OL] (Institute of Applied Linguistics, 2009) by three candidates for the doctoral degree in psychology. These words were divided into four groups with approximately the same amount of words, and the 422 previously mentioned participants completed the assessment at different times. A list of words was presented on the screen one-by-one (programmed in E-prime 1.1), and the participants assigned a value from 1 to 7 according to the word’s threat potential and relevance to examinations. Higher values indicate that the words were more threatening (or relevant).

These words were then ranked according to their values on threat and exam relevance. Those falling into the highest and lowest 10% of their respective score distributions were selected as candidate items. After controlling for frequency and stroke, we obtained 64 two-character Chinese words. The various questionnaires listed previously were performed in the 3 weeks before the final exams, except for the measurement on state anxiety. In order to assess the test–retest reliability of the 64-items vocabulary, a sample of 79 college students (26 males), aged 18–23 years (*M* = 19.96, *SD* = 1.13), returned to complete the assessment of the words after 2 weeks.

#### The Modified ANT

##### Stimuli, apparatus, and design

The stimuli consisted of a row of five visually presented horizontal rectangles, with arrowheads pointing leftward or rightward. The combination of arrowheads and rectangles yielded hollow arrows. The central arrow (leftward or rightward) was considered the target and was flanked by two arrows on each side that could point in the same direction as the target (congruent trials) or in the opposite direction (incongruent trials) or by rectangles (neutral condition; see the three flanker conditions in **Figure 1B**). These five-items combos covered an area of 10 mm × 6 mm. The efficiency of executive attention was indexed by deterioration in reaction time (RT) in incongruent trials compared to congruent trials.

Within the centrally presented hollow arrows, one of the 64 two-character Chinese words, which varied in terms of valence (threat versus non-threat) and relevance to examinations (relevant versus irrelevant), was presented as filler (see **Figure 1C** and **Table 2**). For example, “sweater” was considered an irrelevant and non-threat filler; “ferocious,” “dictionary,” and “exam” were considered irrelevant and threat, relevant and non-threat, and relevant and threat fillers, respectively. The filler word was set in Song typeface with a font size of 9 points and changed randomly between trials.

**Table 2:** Characteristics of the filler words (mean and SD).

Emotionality (*N*)	Nouns (*N*)	Verbs (*N*)	Adjectives (*N*)	Relevance	Threat	Frequency	Strokes
Irrelevant and non-threat(16)	11	2	3	1.55 (0.18)	1.29 (0.20)	33.50 (22.33)	17.38 (3.91)
Irrelevant and threat(16)	3	4	9	1.77 (0.18)	2.86 (0.24)	33.50 (21.62)	17.31 (5.56)
Relevant and non-threat(16)	9	5	2	3.48 (0.40)	1.37 (0.18)	33.25 (28.63)	17.12 (4.23)
Relevant and threat(16)	3	10	3	3.65 (0.58)	2.94 (0.28)	33.62 (35.09)	17.81 (4.64)

In some trials, there were asterisk cues before the stimulus presentation, indicating when or where the target would occur, thereby providing a basis for the participant to direct attention to the cued location (Posner, 1980). The cue conditions (no-cue, central-cue, double-cue, and spatial-cue condition) which have been described in Fan et al. (2002), are illustrated in **Figure 1A**. For the no-cue (no asterisk appeared, participants saw only the fixation point for 100 ms), center-cue (asterisk appeared superimposed over the fixation point), and double-cue trials (asterisks appeared both above and below the fixation point), the target locations were always uncertain. The cue was only valid for the spatial-cue trials (asterisk appeared either above or below the fixation point), which meant that it was at the target position. The efficiency of alerting attention was indexed by improvement in mean RT in double-cue trials compared to no-cue trials. The efficiency of orienting attention was indexed by improvement in mean RT in spatial-cue trials compared to center-cue trials.

Forty-eight conditions (4 cue conditions × 3 flanker types × 2 relevance conditions × 2 threat conditions) were assigned to the repeated testing conditions, with the assignment counterbalanced between experimental (high-anxiety participants) and control group. All four cue conditions were equally probable in the task, as were all three flanker types. Targets appeared above and below the fixation point with equal probability. The words varying in valence and relevance and were randomly distributed among the different cue conditions and flanker types. Group condition was the between-subject factor; the within-subject factors included cue, flanker, and filler conditions. The generation of the stimuli and collection of responses were controlled using the E-prime 1.1 software (Schneider et al., 2002) running under Windows XP on a PC with a 1.6 GHz processor and displayed on a 17-inch flat-panel screen (resolution of 1024 × 768 pixels and refresh rate of 85 Hz).

##### Procedures

Subjects sat about 90 cm away from the screen in a silent and dimly illuminated room, and their heads were stabilized by the headrest of an armchair. Each trial began with a fixation point presented for 400–1,600 ms in the center of the screen. A cue was presented for 100 ms, and 400 ms later, a stimulus-combination was presented. Subjects were asked to identify the direction of the central arrow by pressing the left mouse button for the left direction and the right mouse button for the right direction. They were instructed to respond as quickly and accurately as possible.

The stimulus-combination was presented for up to 1,700 ms, or until the subject responded. The total duration of each trial was 4,000 ms. The fixation point appeared at the same location during the whole trial. The stimulus-combination, which subtended an area of 3.18° × 0.38° of visual angle, was presented 0.25° above or below the fixation point. The cue was presented at similar locations except for the central cue condition. The experimental process is shown in **Figure 1D**. The experiment consisted of a 24-trial full-feedback practice block (RT, whether answer was correct, and cumulative success rate) followed by three blocks of 96 feedback-free trials each (4 cue conditions × 2 combo locations × 2 target directions × 3 flanker types × 2 repetitions), with a 2-min break between blocks. The A-State subscale of the STAI was completed either before or after the revised ANT, with the order counterbalanced between groups. The tasks were completed individually 1 week before the final exams and usually lasted about 40–50 min.

#### Analysis

The questionnaire scores were compared between groups using Fisher’s exact test. The data for the 64 words were analyzed by a 2 (relevance condition) × 2 (threat condition) analysis of variance (ANOVA) with relevance-rating and threat-rating scores serving as dependent variables. In addition, a classical one-way ANOVA of word frequency and stroke was carried out on these different word groups.

We followed the method of Fan et al. (2002) to analyze the effects of cues, flankers, and groups on choice RTs. Two participants were excluded from the analysis for outlier RTs in more than 20% trials (Z-score > 3). Subsequently, 38 participants were screened to remove trials (1.58%) with responses faster than 200 ms and trials with RTs 3 SD greater than an individual’s mean. The remaining RT data from the correct trials (95.56%) were pooled as a function of cue and flanker condition. We carried out a 2 (group condition) × 4 (cue condition) × 3 (flanker type) × 4 (filler-emotion condition) mixed ANOVA of the RT data.

To examine the effect of emotions on attention, we reconstructed the current formula for calculating the efficiency of the attentional network (Fan et al., 2002) by computing the RT difference between the emotional targets and neutral targets (i.e., targets contained irrelevant and non-threat words). The formulae for calculating the alerting effect under different emotional conditions were as follows: Alerting_neutral_ = RT_no-cue,neutral_ – RT_double-cue,neutral_ representing the benefit of the target response speed because of presence versus absence of cues without spatial information in neutral trials. Alerting_irrelevant and threat_ = RT_no-cue,neutral_ – RT_double-cue,irrelevant_, _and threat_ representing the benefit of the target response speed because of alerting in irrelevant and threat trials compared to neutral trials. Alerting_relevant and threat_ = RT_no-cue,neutral_ – RT_double-cue,relevant_, _and threat_ representing the benefit of the target response speed because of alerting in relevant and threat trials compared to neutral trials. Alerting_relevant and non-threat_ = RT_no-cue_, _neutral_ – RT_double-cue_, _relevant_, _and non-threat_ representing the benefit of the target response speed because of alerting in relevant and non-threat trials compared to neutral trials.

The formulae for calculating the orienting effect under different emotional conditions were as follows: Orienting_neutral_ = RT_central-cue,neutral_ – RT_spatial-cue,neutral_ representing the benefit of the target response speed because of presence versus absence of cues with spatial information in neutral trials. Orienting_irrelevant and threat_ = RT_central-cue,neutral_ – RT_spatial-cue,irrelevant_, _and threat_ representing the benefit of the target response speed because of orienting in irrelevant and threat trials compared to neutral trials. Orienting_relevant and threat_ = RT_central-cue,neutral_ – RT_spatial-cue,relevant_, _and threat_ representing the benefit of the target response speed because of alerting in relevant and threat trials compared to neutral trials. Orienting_relevant and non-threat_ = RT_central-cue,neutral_ – RT_spatial-cue,relevant_, _and non-threat_ representing the benefit of the target response speed because of alerting in relevant and non-threat trials compared to neutral trials.

The formulae for calculating the conflict effect under different emotional conditions were as follows: Conflict_neutral_ = RT_incongruent,neutral_ – RT_congruent,neutral_ representing the cost of the target response speed because of incongruence versus congruence in neutral trials. Conflict_irrelevant and threat_ = RT_incongruent,irrelevant_, _and threat central_ – RT_congruent,neutral_ representing the cost of the target response speed because of conflict in irrelevant and threat trials compared to neutral trials. Conflict_relevant and threat_ = RT_incongruent,relevant_, _and threat_ – RT_congruent,neutral_ representing the cost of the target response speed because of conflict in relevant and threat trials compared to neutral trials. Conflict_relevant and non-threat_ = RT_incongruent,relevant_, _and non-threat_ – RT_congruent,neutral_ representing the cost of the target response speed because of conflict in relevant and non-threat trials compared to neutral trials. These differences indicate that the higher the value of the alerting and orienting effect, the more efficient the attentional network is. When it comes to the conflicting effect, the reverse is true (Fan et al., 2002).

We suspected that there might be a close relationship between anxiety sensitivity and depression (Goldenberg et al., 1996; Taylor et al., 1996; Weems et al., 1997), and that trait and state anxiety levels might modulate individual attentional network efficiency (Pacheco-Unguetti et al., 2010). We hence carried out three separate 2 (group condition) × 2 (filler relevance condition) × 2 (filler valence condition) analyses of covariance (ANCOVAs) by entering all three covariates (trait anxiety, anxiety, and depression scores), with alerting, orienting, and conflicting effects as dependent variables. All the data were analyzed using SPSS 16.0 for Windows.

### Results and Discussion

#### Questionnaire Results

As shown in **Table 1A**, test-anxious subjects in the revised ANT had significantly greater TA [*t*_(39)_ = 15.09, *p* < 0.001] than controls. The same result was obtained for the subscales of the STAI [trait *t*_(39)_ = 4.54, *p* < 0.001; state *t*_(39)_ = 4.49, *p* < 0.001]. These findings are consistent with the notion that describes TA as a situation specific anxiety (Spielberger and Vagg, 1987). Furthermore, test-anxious subjects had significantly greater depression levels than controls [*t*_(39)_ = 4.77, *p* < 0.001]. This result confirmed the close relationship between anxiety sensitivity and depression (Goldenberg et al., 1996; Taylor et al., 1996; Weems et al., 1997).

#### Filler Word Results

The results of the vocabulary assessment are shown in **Table 2** and reveal a main effect of relevance [*F*_(1,420)_ = 415.07, *p* < 0.001, $\eta_{p}^{2}$ = 0.87], with rating levels significantly greater for test-relevant than test-irrelevant words, as well as a main effect of valence [*F*_(1,420)_ = 759.76, *p* < 0.001, $\eta_{p}^{2}$ = 0.93], with rating levels significantly greater for threat than non-threat words. Thus, the four different word groups indeed varied in either relevance or valence. It is also important to note that there were no significant differences in word frequency [*F*_(1,421)_ = 0.00, *p* = 1.00] and stroke [*F*_(1,421)_ = 0.06, *p* = 0.98] between the different word groups.

The intra-scale reliability of each conceptual domain was determined by computing Cronbach’s alpha coefficients. Values of 0.94 (for relevance) and 0.94 (for valence) were taken as indicating satisfactory reliability. Test–retest repeatability correlation coefficients for the two domain scores were highly significant (*p* < 0.001). Cronbach’s alpha coefficients were 0.81 and 0.83 for the relevance and valence subscale, respectively.

#### Effects of Cues, Flankers, and Fillers on Choice Reaction Times

**FIGURE 2:**
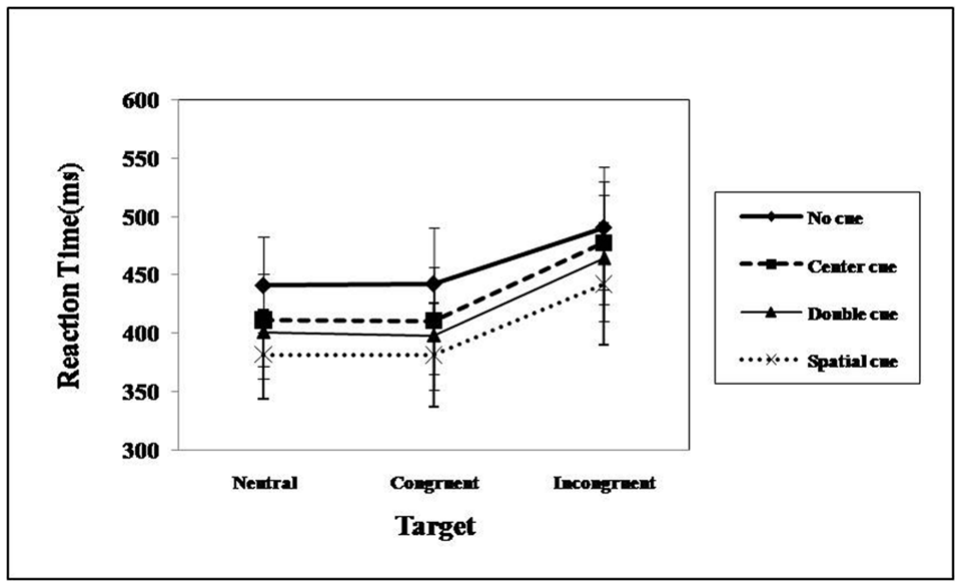
**Mean RT from correct trials as a function of cue and flanker condition under emotional distraction**.

**Table 3** summarizes RT data pooled from correct trials as a function of cue, flanker, and filler-emotion condition. The results replicated the findings of Fan et al. (2002). Statistically significant differences were found for the cue [*F*_(3,34)_ = 122.52, *p* < 0.001, $\eta_{p}^{2}$ = 0.77] and flanker [*F*_(2,35)_ = 508.98, *p* < 0.001, $\eta_{p}^{2}$ = 0.93] main effects and for their interaction [*F*_(6,31)_ = 5.04, *p* < 0.001, $\eta_{p}^{2}$ = 0.12; see **Figure 2**]. The simple effect analysis suggested that, regardless of the cue condition, subjects took more time to react under incongruent flankers, and this effect was enhanced under the alerting cue conditions (central and double cues). Considering that both cue and flanker effects are in line with the principles of the ANT design, attentional network functions can be thus formulated (Fan et al., 2002).

**Table 3:** Reaction time (RT) data (mean and SD) under each condition.

Flanker	Filler emotion	Group	Cue condition				
			No cue	Center cue	Double cue	Spatial cue	Total
Congruent	IRNT	Control	439 (48)	436 (33)	390 (49)	384 (53)	411 (43)
		Experiment	448 (54)	413 (36)	394 (43)	390 (49)	410 (45)
	IRT	Control	448 (50)	441 (54)	392 (56)	378 (52)	408 (48)
		Experiment	452 (59)	415 (61)	406 (57)	394 (56)	416 (50)
	RNT	Control	444 (60)	443 (44)	396 (57)	376 (50)	407 (52)
		Experiment	450 (56)	411 (36)	411 (46)	387 (43)	414 (46)
	RT	Control	445 (46)	479 (41)	400 (54)	380 (50)	406 (46)
		Experiment	447 (41)	424 (49)	403 (50)	396 (45)	414 (40)
Incongruent	IRNT	Control	505 (54)	484 (48)	469 (49)	455 (55)	478 (52)
		Experiment	506 (56)	498 (51)	470 (41)	466 (52)	489 (49)
	IRT	Control	494 (75)	506 (63)	469 (54)	445 (68)	479 (59)
		Experiment	547 (70)	558 (62)	542 (49)	503 (60)	503 (51)
	RNT	Control	506 (63)	494 (51)	461 (54)	445 (50)	478 (52)
		Experiment	526 (63)	505 (54)	499 (47)	482 (48)	497 (45)
	RT	Control	494 (51)	444 (52)	479 (56)	461 (58)	478 (50)
		Experiment	520 (51)	527 (41)	520 (59)	502 (51)	496 (46)
Neutral	IRNT	Control	444 (52)	440 (42)	402 (46)	380 (42)	416 (44)
		Experiment	440 (46)	422 (49)	409 (33)	393 (46)	415 (41)
	IRT	Control	445 (51)	440 (45)	392 (50)	372 (45)	412 (49)
		Experiment	455 (46)	419 (54)	413 (41)	397 (45)	421 (44)
	RNT	Control	441 (47)	438 (35)	409 (47)	378 (47)	417 (42)
		Experiment	455 (45)	417 (36)	403 (36)	392 (40)	417 (37)
	RT	Control	439 (37)	414 (58)	395 (49)	376 (40)	406 (42)
		Experiment	438 (44)	416 (49)	402 (44)	386 (34)	411 (39)

*IRNT, irrelevant and non-threat; IRT, irrelevant and threat; RNT, relevant and non-threat; RT, relevant and threat.*

The results of the ANOVA showed no significant main effect of group [*F*_(1,36)_ = 0.91, *p* = 0.35]. However, the ANOVA revealed a significant filler-emotion main effect [*F*_(3,34)_ = 10.89, *p* < 0.001, $\eta_{p}^{2}$ = 0.23]. This effect was qualified by a significant group × filler-emotion interaction [*F*_(3,34)_ = 12.58, *p* < 0.001, $\eta_{p}^{2}$ = 0.26]. Contrasting high and low TA groups showed that targets embedded with emotional (i.e., irrelevant and threat, relevant and non-threat, and relevant and threat) versus neutral words prolonged the RT only in the TA group, suggesting that controls were free from the emotional interference. The effect of filler-emotion was also qualified by a significant group × filler-emotion × cue × flanker interaction [*F*_(18,19)_ = 1.70, *p* < 0.05, $\eta_{p}^{2}$ = 0.05]. Further analysis showed that students with TA took more time for dealing with targets embedded with irrelevant and threat words in conflict trials under center-cue [*F*_(1,36)_ = 6.63, *p* < 0.05, $\eta_{p}^{2}$ = 0.16] and no-cue [*F*_(1,36)_ = 5.17, *p* < 0.05, $\eta_{p}^{2}$ = 0.13] conditions, compared to controls. The same was true for irrelevant and threat [*F*_(1,36)_ = 7.52, *p* < 0.01, $\eta_{p}^{2}$ = 0.17], relevant and non-threat [*F*_(1,36)_ = 5.26, *p* < 0.05, $\eta_{p}^{2}$ = 0.13], and relevant and threat [*F*_(1,36)_ = 5.13, *p* < 0.05, $\eta_{p}^{2}$ = 0.13] fillers in conflict trials under spatial-cue conditions. Similarly, in the double-cue conditions, the corresponding ANOVA values were as follows: *F*_(1,36)_ = 18.48, *p* < 0.001, $\eta_{p}^{2}$ = 0.34; *F*_(1,36)_ = 5.23, *p* < 0.05, $\eta_{p}^{2}$ = 0.13; *F*_(1,36)_ = 4.91, *p* < 0.05, $\eta_{p}^{2}$ = 0.12.

#### Efficiency of Attentional Networks

**Table 4** shows the efficiency of subjects’ alerting, orienting, and executive attention networks in these emotional tasks. There were no significant main effects of group [*F*_(1,33)_ = 2.91, *p* = 0.10], relevance [*F*_(1,33)_ = 0.05, *p* = 0.82], or valence [*F*_(1,33)_ = 1.80, *p* = 0.19] in the efficiency of alerting. No significant interactions (all four *F*’s < 1.14) were found. We found similar results for the efficiency of orienting; the corresponding *F*-values for group, relevance, and valence were 1.02 (*p* = 0.32), 1.00 (*p* = 0.32), and 0.74 (*p* = 0.40), respectively. Again, there were no significant interactions (all four *F*’s < 1.48). These results suggest that despite the emotionality of the targets, there was no difference in alerting or orienting efficiency between test-anxious and control individuals.

**Table 4:** Alerting, orienting, and executive effects (mean RTs and SD) under emotional distraction.

Index	Alerting (ms)				Orienting (ms)				Executive (ms)			
	IRNT	IRT	RNT	RT	IRNT	IRT	RNT	RT	IRNT	IRT	RNT	RT
Control (*M*)	43.68	46.51	46.83	36.04	35.75	36.16	43.40	36.10	67.57	68.32	67.56	67.37
Control (*SD*)	27.94	28.51	29.76	23.70	26.23	33.24	26.61	30.13	24.61	26.97	25.53	21.46
Experiment (*M*)	39.05	37.92	39.09	41.59	29.74	26.26	26.87	30.90	78.94	93.77	87.62	86.24
Experiment (*SD*)	39.01	29.96	26.76	34.59	29.09	34.70	29.19	27.20	24.92	34.64	30.41	28.94

*IRNT, irrelevant and non-threat; IRT, irrelevant and threat; RNT, relevant and non-threat; RT, relevant and threat.*

**FIGURE 3:**
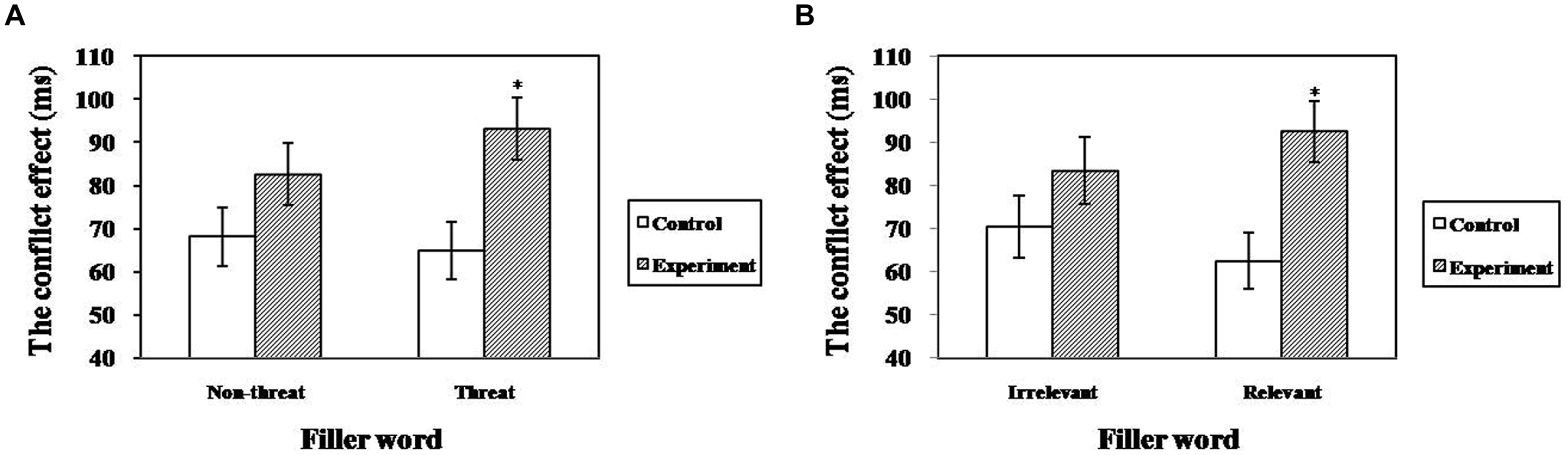
The interaction between group and valence/relevance for executive attention in the revised ANT (*p < 0.05). (A) Interaction between group and valence, **(B)** Interaction between group and relevance.

The ANCOVA for executive attention revealed a significant main effect of relevance [*F*_(1,33)_ = 9.08, *p* < 0.01, $\eta_{p}^{2}$ = 0.22) and a marginally significant main effect of group [*F*_(1,33)_ = 3.69, *p* = 0.06, $\eta_{p}^{2}$ = 0.10]; however, no main effect of valence was observed [*F*_(1,33)_ = 0.67, *p* = 0.42]. Importantly, these effects were qualified by a significant group × valence interaction [*F*_(1,33)_ = 4.41, *p* < 0.05, $\eta_{p}^{2}$ = 0.12] and a marginally significant group × relevance interaction [*F*_(1,33)_ = 3.63, *p* = 0.06, $\eta_{p}^{2}$ = 0.10]. The simple effect analysis revealed that the TA group (*M* = 93.20 ± 7.14) was more impaired on executive attention than controls (*M* = 64.97 ± 6.63) when the fillers were threat words compared with non-threat words (see **Figure 3A**). The same was true when the filler words were test-relevant (*M*_TA_ = 92.42 ± 7.13 versus *M*_controls_ = 62.53 ± 6.14; see **Figure 3B**). These results suggest that test-anxious individuals showed lower efficiency of executive attention than controls especially when they were exposed to a situation involving threat/test-related information.

To validate the effect of the modified ANT, we introduced Experiment 2 in which the original ANT was employed. Considering the findings from Experiment 1 and the evidence indicating that elementary cognitive operations may be intact in individuals with anxiety and depression in the conditions that are free from emotional distracters (Airaksinen et al., 2005; Pardo et al., 2006; Preiss et al., 2010), we expected that the revised task would be more sensitive to TA than the original ANT when measuring executive attention.

Hypothesis 2: Emotional distraction (i.e., revised) task is expected to reduce executive rather than alerting/orienting attention in contrast to distraction-free (i.e., original) task in high versus low test-anxious students.

## Experiment 2

### Methods

#### Participants

Another 40 (19 males) students of the subject pool of 422 original participants (see **Table 1B**) were assigned to the original ANT. The basic requirements for their participation were the same as those for Experiment 1.

#### Measures

Measures were the same as those in Experiment 1.

#### The Original ANT

##### Stimuli, apparatus, and design

**FIGURE 4:**
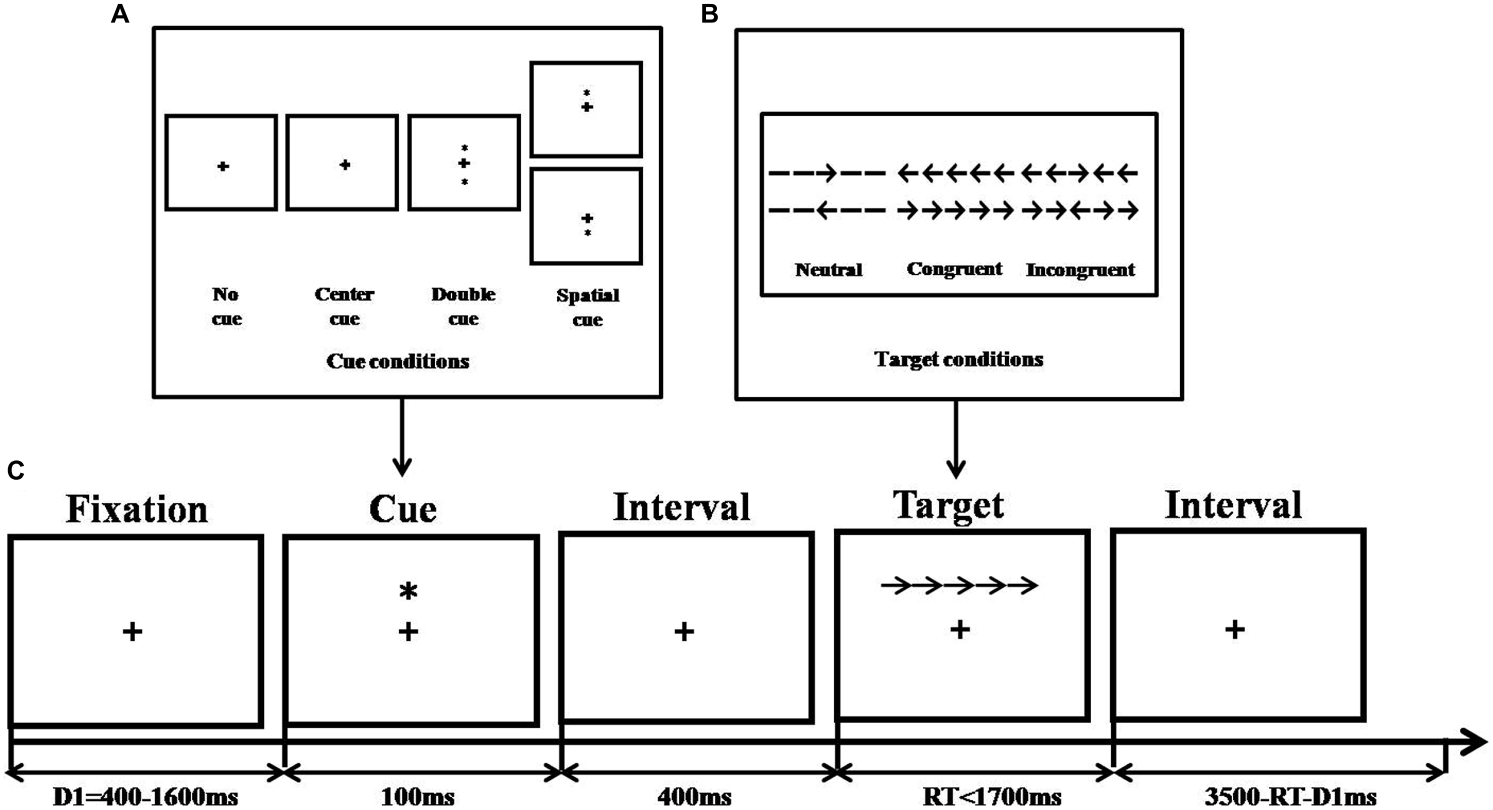
**Experimental procedure of original ANT (adapted from Fan et al., 2002). (A)** Cue conditions, **(B)** Stimuli without emotion interference, and **(C)** An example of the procedure.

Stimuli were different from Experiment 1, for example, black lines replaced rectangles, thus yielding solid arrows or arrow-line combinations (see **Figure 4**). In addition, no word was used as filler. The combination subtended an area of 3.08° of visual angle and appeared either 1.06° above or below the fixation point. The cue conditions and the apparatus were the same as for the revised ANT. More details are given in Fan et al. (2002).

##### Procedures

The procedure was the same as described by Fan et al. (2002). Briefly, participants viewed the stimuli from a distance of 65 cm under four cue conditions and two flanker conditions. They were instructed to identify the direction of the centrally presented arrow and to respond as fast and as accurately as possible. Responses were collected via two mouse buttons. A session consisted of a 24-trial practice block and three experimental blocks. Each experimental block consisted of 96 trials (48 conditions, as described in Experiment 1, with two repetitions). The presentation of trials was in a random order. Again, the A-State subscale of the STAI was completed either before or after the revised ANT, with the order counterbalanced between groups.

#### Analysis

Questionnaire analyses were the same as those in Experiment 1. We followed the method of Fan et al. (2002) to calculate efficiency of executive attention based on the RT data from the original ANT. The formulae were as follows: alerting effect = RT_no-cue_ – RT_double-cue_, orienting effect = RT_center-cue_ – RT_spatial-cue_, conflicting effect = RT_incongruent_ – RT_congruent_. After consolidating the data on efficiency of attentional networks, which derived from the original and revised ANT (Experiment 1), we then carried out three separate 2 (task type) × 2 (group condition) ANCOVAs by entering all three covariates (trait anxiety, anxiety, and depression scores), with alerting, orienting, and conflicting effects as dependent variables.

### Results and Discussion

#### Questionnaire Results

We observed similar results in demographic characteristics of the subjects compared to those of Experiment 1. As shown in **Table 1B**, test-anxious subjects had significantly higher levels of TA [*t*_(38)_ = 12.40, *p* < 0.001], trait anxiety [*t*_(38)_ = 4.99, *p* < 0.001], state anxiety [*t*_(38)_ = 3.74, *p* < 0.001], and depression [*t*_(38)_ = 6.30, *p* < 0.001] than controls.

#### The Effects of the Revised ANT

**FIGURE 5:**
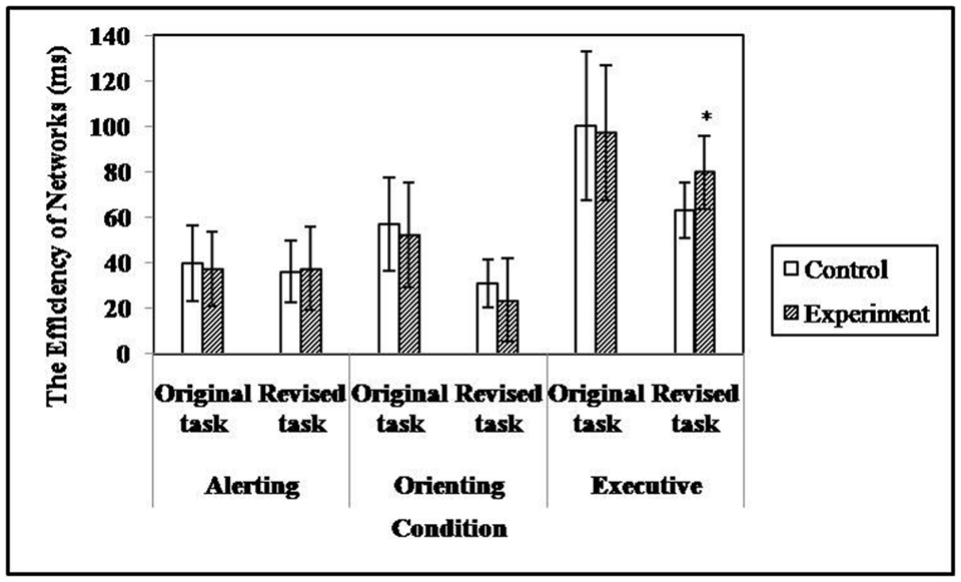
The efficiency of attentional networks in different tasks (*p < 0.05).

**Figure 5** shows the efficiency of subjects’ alerting, orienting, and executive attention in the different tasks. Although no main effect of group [*F*_(1,71)_ = 3.21, *p* = 0.08] was found for the efficiency of executive attention, the ANCOVA yielded a significant main effect of task type [*F*_(1,71)_ = 20.30, *p* < 0.001, $\eta_{p}^{2}$ = 0.22] and a significant interaction between group and task type [*F*_(1,71)_ = 4.20, *p* < 0.05, $\eta_{p}^{2}$ = 0.06]. The simple effect analysis suggested that the efficiency of executive attention in high TA individuals (*M* = 85.34 ± 6.66) was significantly lower than that of controls (*M* = 59.38 ± 6.09) in the revised task, while no difference between groups was found in the original task (*M*_TAgroup_ = 99.23 ± 6.07 versus *M*_controls_ = 96.64 ± 6.38). The results indicated that the adaptation of the ANT enhanced its sensitivity to TA when measuring executive attention.

There was no significant group main effect [*F*_(1,71)_ = 0.05, *p* = 0.82] or interaction between task type and group [*F*_(1,71)_ = 0.32, *p* = 0.57] for the efficiency of alerting attention. The same was true when applying the analysis to the efficiency of orienting attention; the corresponding ANCOVA values were *F*_(1,71)_ = 0.01, *p* = 0.92 and *F*_(1,71)_ = 0.02, *p* = 0.90. In addition, no significant difference was found between groups when comparing the efficiency scores of executive [*F*_(1,71)_ = 0.07, *p* = 0.79], alerting [*F*_(1,71)_ = 0.28, *p* = 0.60], or orienting [*F*_(1,71)_ = 0.02, *p* = 0.88] attention that derived from the original task. The results indicated that performance in the original ANT was insensitive to the level of TA.

## General Discussion

As we expected, test-anxious students showed a significant deficit in the executive attention network when they were faced with threat/test-related distracters (Experiment 1). This result is consistent with event-related potential (ERP) findings, indicating that the processing of negative emotions was linked to decrements in executive attention in the high trait-anxiety group (Dennis and Chen, 2007). The current result also supports findings of a previous study using a modified Stroop color-naming task, which found that test-anxious individuals took longer to respond to test-threat words compared to controls (Lawson, 2006). In fact, various studies employing different tasks, which tapped different aspects of control processing, also reached the conclusion that threat-related information negatively affected executive function of anxious individuals (Bishop et al., 2004; Wieser et al., 2009); considering that effortful control, especially behavioral or attentional control, is strongly related to the construct of executive function (Muris and Ollendick, 2005).

Both Experiments 1 and 2 suggested that impaired executive attention in test-anxious students was only related to a specific situation, which involved emotional, in particular threat/test-related distracters. This may be due to the poor stability of the attention bias in different situations (Bar-Haim et al., 2007). For example, individuals with trait anxiety were more sensitive to threat stimuli in stressful situations (Mogg et al., 1997), and state anxiety was related to the attentional narrowing of negative affect (Rowe et al., 2007). In the present study, incongruent flanker displays represent the high-conflict condition (congruent flanker displays, in contrast, represent the low conflict conditions), and conflict is associated with stress (Mann, 1992; Dehais et al., 2012). Moreover, time pressure might also decrease the efficiency of attentional allocation (Assink et al., 2015). On these grounds, we speculate that the overlapping effects of the flanker conflict and the resource competition between targets and fillers under time pressure (upper limit of 1,700 ms) increased the stress level in conflict conditions compared with non-conflict conditions. Compared to the controls, test-anxious students perceived more stress or higher pressure, which triggered a more distinct attentional bias toward threat/test-related distracters or evoked attentional narrowing of negative emotions. This consequently caused delayed identification of the targets in conflict trials, which was reflected as executive impairment in test-anxious students compared to controls.

Another possible explanation stems from the study of Mathews and MacLeod (2005), which demonstrated that anxious people apply a top–down activation strategy due to vigilance but have difficulty inhibiting the unexpected stimulus-related bottom–up activation. On the one hand, it has been found that the goal-directed attentional system (i.e., top–down activation) could regulate the stimulus-driven attentional system, for example, a threat bias could be moderated by attentional control (Derryberry and Reed, 2002), which decreased the disruptive effect of negative cues (Cohen et al., 2011). Accordingly, the application of top–down activation strategies may have regulated the impact of threat distracters (i.e., reduced attentional bias toward threats) on alerting/orienting attention, which is primarily related to vigilance, in test-anxious students.

On the other hand, the response to multiple stress conditions (due to the design of our modified ANT) during conflict trials might not only deplete the self-control resources in test-anxious students under emotional distraction, but might make it hard to replenish resources as well, hence weakening the inhibitions for negative distraction during task performance (Cohen et al., 2011). Alternatively, the depleted self-control resources could make it difficult to suppress the “pop-out” effect induced by unintended threat distraction (Bertrams et al., 2013), as “bottom–up” sensory driven mechanisms were active at the moment. Therefore, TA subjects’ target identification was delayed to a greater degree by threat/test-related distracters in incongruent trials compared with congruent trials, and compared to target identification of controls. These findings are in accordance with the attentional control theory, which proposes that anxiety decreases the influence of the goal-directed attentional system and increases the influence of threat distraction related to the stimulus-driven attentional system (Eysenck et al., 2007), and provide further evidence that “anxiety is associated with reduced top–down control over threat-related distracters” (Bishop et al., 2004, p. 184).

The results that threat/test-related distracters had no impact on alerting/orienting attention in TA students (Experiment 1) did not meet our expectations. One reason may be that anxious subjects, as we previously mentioned, apply a top–down activation strategy due to vigilance. Moreover, we speculate that processing patterns differed from those of the executive attention network when assessing alerting or orienting networks. Because two-thirds of the trials were non-conflict trials in the latter two assessment models, test-anxious students suffered less stress at this time in contrast to measuring conflict effects, thus were not more sensitive to threat stimuli than controls under these conditions. Consequently, no difference in RT data (on target identification) was observed between controls and highly test-anxious students. In fact, these results support the findings that threat distraction neither enhanced the orienting attention in a state anxiety study (Dennis et al., 2008) nor had an impact on alerting attention in a trait anxiety study (Dennis and Chen, 2007), although the precise nature of the impact of threat distraction on orienting or executive attention seemed rather complicated in the latter study. Taken together, these findings reveal the complexity of attention processing in test-anxious individuals under threatening or evaluative situations: such individuals share certain characteristics with those suffering from trait and state anxiety while maintaining certain differences; thus, confirming the concept of TA that combines attributes from both trait and state anxiety (Spielberger and Vagg, 1987; Hodapp et al., 1995).

Experiment 2 showed that the revised ANT was more sensitive to TA than the original ANT when measuring executive attention. We speculate that emotional distracters play a critical role in the regulation of executive function in TA students. One reason may be that emotional distracters provided conditions to induce an attentional bias or the preemption of resources required for emotion processing, compared to conditions without distracters. Considering that the performance on the original ANT is insensitive to the level of TA, the results from Experiment 2 indicate that the attentional deficit in test-anxious individuals represents a situation-related defect of a single component of attention, rather than an underlying structural and universal deficit of attention.

There are still many gaps in our knowledge about the stage of information processing in which attentional biases occur (Fox et al., 2000; Amir et al., 2003; Mogg et al., 2004; Koster et al., 2005; Cisler and Koster, 2010), and there are many debates about the mechanisms that mediate the biases (Cisler and Koster, 2010; Putwain et al., 2011). In the present study, threat/test-related distracters did not affect alerting/orienting attention in TA individuals, indicating that the bias may not occur at the early stage (detection). In contrast, threat/test-related distracters did affect executive attention in TA individuals, indicating that the bias may occur at the late stage (execution). The findings that threat/test-related stimuli modulate executive attention in TA individuals may tap into the mechanism of bias modulation and at least partly explain why TA individuals have difficulty to disengage attention from threat/test-related distracters (Vasey et al., 1996; Keogh and French, 2001). Considering that the bias might depend on the interaction between bottom–up activation of threat representations by a threat-detection system and top–down activation of competing representations related to other goals by an attentional control system (Mathews and MacLeod, 2005), our findings strengthen the notion that executive attention may be the key structure to the understanding of attentional biases toward threat-related material in anxious subjects (Avila and Parcet, 2002).

The present study has several limitations that need to be acknowledged. First of all, TA was measured as a factor between subjects by self-report, and this criterion may be involved in other personal characteristics such as learning ability (Zeidner, 1998), self-esteem (Lekarczyk and Hill, 1969), and social desirability bias (Tanaka-Matsumi and Kameoka, 1986). Thus, effects from these characteristics cannot be convincingly excluded. In future studies, a within-subject design, in which TA is elicited by emotional stimuli, might be introduced to control for demand characteristics across each condition. Secondly, the way we introduced emotional distraction in the revised ANT consisted of embedding emotional two-character Chinese words in hollow arrows. Although the simultaneous presentation of target and distraction in spatial terms led to a competition for attentional resources, the ecological validity of the measurement is ambiguous due to the artificial interference. The use of more ecological emotion manipulations is recommended, for instance, by integrating images with sound in a cross-modal stimulus presentation. Lastly, our findings were mainly derived from behavioral evidence. ERPs on the other hand would provide a powerful tool for capturing aspects of emotion-attention interactions that behavioral studies alone might miss (Dennis and Chen, 2007); thus, further studies should concentrate on the neural mechanisms of the impaired attentional network in test-anxious individuals.

Despite these limitations, we believe that the present study contributes to the understanding of attentional mechanisms in TA individuals. To our knowledge, the study provides the first investigation of the attentional system in test-anxious individuals under emotional distraction and adds to our knowledge of vulnerability to threat-related stimuli in test-anxious individuals. In addition, the study developed a revised ANT, in which an emotional factor was integrated into the target yielding a more sensitive measurement of TA. This adaptation offers a novel method, which can simultaneously examine the “real-time” impact of emotional processing on attention performance in multiple domains. The revised ANT may contribute to the development of future work to serve as validation assessment of cognitive training in TA.

## Conflict of Interest Statement

The authors declare that the research was conducted in the absence of any commercial or financial relationships that could be construed as a potential conflict of interest.
