# Supplementary material for: Modulation of executive attention by threat stimulus in test-anxious students
Source: Front Psychol. 2015 Oct 1;6:1486. doi: 10.3389/fpsyg.2015.01486 (PMC4589644; doi:10.3389/fpsyg.2015.01486)
Supplement: Supplementary file 1 [file Table_1.DOCX]

APPENDIX

Vocabulary(in Chinese) of Experiment 1

| No | Word | Emotion | Relevance | Valence | Frequency | Strokes |  | No | Word | Emotion | Relevance | Valence | Frequency | Strokes |
| --- | --- | --- | --- | --- | --- | --- | --- | --- | --- | --- | --- | --- | --- | --- |
| 1 | 绿地 | IRNT | 1.39 | 1.00 | 49 | 17 |  | 33 | 算式 | RNT | 3.66 | 1.69 | 18 | 20 |
| 2 | 公交 | IRNT | 1.72 | 1.57 | 26 | 10 |  | 34 | 优等 | RNT | 3.67 | 1.38 | 15 | 18 |
| 3 | 报销 | IRNT | 1.53 | 1.38 | 27 | 19 |  | 35 | 题意 | RNT | 3.98 | 1.54 | 10 | 25 |
| 4 | 暖和 | IRNT | 1.57 | 1.01 | 74 | 21 |  | 36 | 尺子 | RNT | 2.98 | 1.28 | 22 | 7 |
| 5 | 领班 | IRNT | 1.57 | 1.34 | 7 | 21 |  | 37 | 获奖 | RNT | 3.47 | 1.25 | 60 | 19 |
| 6 | 毛衣 | IRNT | 1.35 | 1.09 | 31 | 10 |  | 38 | 发奋 | RNT | 3.65 | 1.24 | 10 | 13 |
| 7 | 憨厚 | IRNT | 1.62 | 1.25 | 35 | 24 |  | 39 | 科目 | RNT | 3.98 | 1.60 | 109 | 14 |
| 8 | 发型 | IRNT | 1.23 | 1.39 | 35 | 14 |  | 40 | 博学 | RNT | 3.50 | 1.27 | 13 | 20 |
| 9 | 媒体 | IRNT | 1.73 | 1.48 | 7 | 19 |  | 41 | 出勤 | RNT | 3.23 | 1.64 | 25 | 18 |
| 10 | 酒壶 | IRNT | 1.46 | 1.38 | 20 | 20 |  | 42 | 课桌 | RNT | 3.14 | 1.33 | 15 | 20 |
| 11 | 行情 | IRNT | 1.84 | 1.31 | 60 | 17 |  | 43 | 钢笔 | RNT | 3.20 | 1.17 | 54 | 19 |
| 12 | 挂念 | IRNT | 1.78 | 1.46 | 20 | 17 |  | 44 | 文具 | RNT | 3.30 | 1.24 | 27 | 12 |
| 13 | 零头 | IRNT | 1.61 | 1.29 | 5 | 18 |  | 45 | 荣获 | RNT | 3.19 | 1.16 | 75 | 19 |
| 14 | 留恋 | IRNT | 1.70 | 1.47 | 76 | 20 |  | 46 | 放假 | RNT | 2.92 | 1.23 | 20 | 19 |
| 15 | 纽扣 | IRNT | 1.38 | 1.40 | 18 | 13 |  | 47 | 满分 | RNT | 4.39 | 1.58 | 9 | 17 |
| 16 | 馄饨 | IRNT | 1.32 | 0.89 | 46 | 18 |  | 48 | 字典 | RNT | 3.49 | 1.35 | 50 | 14 |
| 17 | 霸道 | IRT | 1.54 | 2.54 | 6 | 33 |  | 49 | 差错 | RT | 2.73 | 2.60 | 105 | 22 |
| 18 | 嫉恨 | IRT | 1.90 | 3.16 | 7 | 22 |  | 50 | 名次 | RT | 4.42 | 2.57 | 29 | 12 |
| 19 | 丑闻 | IRT | 1.70 | 3.14 | 8 | 13 |  | 51 | 备考 | RT | 4.29 | 2.48 | 2 | 14 |
| 20 | 凶悍 | IRT | 1.84 | 2.82 | 11 | 14 |  | 52 | 焦虑 | RT | 2.86 | 2.85 | 98 | 22 |
| 21 | 惨叫 | IRT | 1.55 | 3.01 | 25 | 16 |  | 53 | 留级 | RT | 3.73 | 3.08 | 5 | 16 |
| 22 | 畏缩 | IRT | 2.09 | 2.49 | 26 | 23 |  | 54 | 答错 | RT | 3.71 | 2.92 | 5 | 25 |
| 23 | 粗鲁 | IRT | 1.76 | 2.57 | 26 | 23 |  | 55 | 怯场 | RT | 3.47 | 3.03 | 4 | 14 |
| 24 | 伤残 | IRT | 1.78 | 3.01 | 28 | 15 |  | 56 | 担忧 | RT | 2.83 | 2.93 | 67 | 15 |
| 25 | 小偷 | IRT | 1.54 | 2.94 | 28 | 14 |  | 57 | 粗心 | RT | 3.12 | 2.67 | 31 | 15 |
| 26 | 狠心 | IRT | 1.75 | 2.94 | 32 | 13 |  | 58 | 倒数 | RT | 3.58 | 2.86 | 26 | 23 |
| 27 | 疯子 | IRT | 1.73 | 2.62 | 41 | 12 |  | 59 | 抄袭 | RT | 3.47 | 3.10 | 33 | 18 |
| 28 | 穷困 | IRT | 1.71 | 2.49 | 43 | 14 |  | 60 | 作弊 | RT | 3.98 | 3.00 | 12 | 21 |
| 29 | 怒吼 | IRT | 1.86 | 2.92 | 50 | 16 |  | 61 | 高考 | RT | 4.44 | 2.88 | 85 | 16 |
| 30 | 耻辱 | IRT | 2.19 | 3.18 | 61 | 20 |  | 62 | 失败 | RT | 3.26 | 3.35 | 11 | 13 |
| 31 | 骗子 | IRT | 1.63 | 2.96 | 70 | 15 |  | 63 | 补考 | RT | 4.20 | 3.29 | 14 | 13 |
| 32 | 无耻 | IRT | 1.73 | 2.96 | 74 | 14 |  | 64 | 落榜 | RT | 4.27 | 3.47 | 11 | 26 |

IRNT: irrelevant & non-threat, IRT: irrelevant & threat, RNT: relevant & non-threat, RT: relevant & threat.
